# Interrelationships between symptom burden and health functioning and health care utilization among veterans with persistent physical symptoms

**DOI:** 10.1186/s12875-020-01193-y

**Published:** 2020-07-01

**Authors:** Dennis Fried, Lisa M. McAndrew, Drew A. Helmer, Sarah Markowitz, Karen S. Quigley

**Affiliations:** 1 Department of Veterans Affairs, NJ War Related Illness & Injury Study Center, 385 Tremont Ave. Mailstop 129, East Orange, NJ 07018 USA; 2 Department of Epidemiology, Rutgers, The State University of New Jersey, 185 South Orange Avenue, MSB, Newark, NJ 07101 USA; 3 New Jersey Medical School, Rutgers, The State University of New Jersey, 185 South Orange Avenue, MSB, Newark, NJ 07101 USA; 4 Wells College, 170 Main St, Aurora, NY 13026 USA; 5 Interdisciplinary Affective Science Laboratory, Northeastern University, 360 Huntington Ave, Boston, MA 02115 USA; 6 Department of Veterans Affairs, Bedford Memorial Hospital, 200 Springs Rd, Bedford, MA 01730 USA

**Keywords:** Medically unexplained symptoms, Persistent physical symptoms, Veterans, Health care utilization, Symptoms, Health functioning

## Abstract

**Background:**

Between 10 and 50% of primary care patients present with persistent physical symptoms (PPS). Patients with PPS tend to utilize excessive or inappropriate health care services, while being stuck in a deleterious cycle of **inactivity, deconditioning, and further worsening of symptoms and disability**. Since military deployment (relative to non-deployment) is associated with greater likelihood of PPS, we examined the interrelationships of health care utilization, symptom burden and functioning among a sample of recently deployed Veterans with new onset persistent physical symptoms.

**Methods:**

This study analyzed a cohort of 790 U.S. soldiers who recently returned from deployment to Iraq or Afghanistan. Data for this analysis were obtained at pre- and post-deployment. We used moderation analyses to evaluate interactions between physical symptom burden and physical and mental health functioning and four types of health care utilization one-year after deployment, after adjusting for key baseline measures.

**Results:**

Moderation analyses revealed significant triple interactions between physical symptom burden and health functioning and: primary care (*F* = 3.63 [2, 303], R^2^Δ = .02, *p* = 0.03), specialty care (*F* = 6.81 [2, 303] R^2^Δ =0.03, *p* < .001), allied therapy care (*F* = 3.76 [2, 302], *R*^*2*^Δ = .02, *p* = 0.02), but not mental health care (*F* = 1.82 [1, 303], *R*^*2*^Δ = .01, *p* = .16), one-year after deployment.

**Conclusions:**

Among U.S. Veterans with newly emerging persistent physical symptoms one-year after deployment, increased physical symptom burden coupled with decreased physical and increased mental health functioning was associated with increased medical care use in the year after deployment. These findings support whole health initiatives aimed at improving health function/well-being, rather than merely symptom alleviation.

## Background

Persistent physical symptoms (PPS) - also known as medically unexplained symptoms (MUS) [1, 2] - is the umbrella term given to chronic physical symptoms common to physical and mental health conditions that have unclear or contested cause [3, 4]. PPS is common, though prevalence estimates vary greatly. General population studies reveal that between 10 and 50% of primary care patients present with persistent physical symptoms (e.g., chronic widespread pain, chronic fatigue syndrome) [5].

In general, patients with PPS, relative to those without PPS, are more likely to: have impaired physical and mental health functioning [6]; receive excessive health care [7]; have higher annual health care costs [2, 6]; consult their physicians more frequently [3, 4, 8]; have longer visits to doctors for treatment of chronic conditions, such as diabetes [9]; receive “disease/symptom-based care” leading to unnecessary laboratory testing/treatment [2, 6, 7]; experience iatrogenic complications [10]; have greater dependency on medical care [10, 11].

Despite the potential for excessive health care and the identified need to reduce such care [5, 12], few quantitative studies have considered key factors that trigger patients with new PPS to seek care. The medical community, providers and researchers often assume that the severity of the PPS and desire to understand the cause of the symptoms are the primary reasons for patients to seek health care. In response, providers report feeling pressured to provide medical intervention and assessment focused on diagnosing underlying disease and reducing symptom severity – escalating health care utilization. In contrast, clinical practice guidelines advise reducing unnecessary medical intervention and assessment and instead focusing on managing PPS to improve health functioning and well-being [3, 4].

There is an assumption that physical symptom burden independently predicts health care utilization [13] - in other words, increased physical symptom burden in and of itself leads to increased health care utilization. There is evidence to suggest, however, that physical symptom burden may work in tandem with physical and mental health functioning to drive health care utilization [14]. For example, no more than half and as little as one-tenth of primary care patients seek care when they experience physical symptoms [4, 5]; that is, most of the time physical symptoms do not lead to health care utilization. When patients do seek health care, studies reveal that they talk with their provider more about improving health function than simply reducing symptom severity [15], suggesting patients may be triggered to seek health care by decrements in health function.

Leventhal and colleagues [14] common-sense model of self-regulation, proposes that people develop heuristics or rules of thumb that guide their interpretation of symptoms. In general, symptoms that are severe, persistent or new are more likely than symptoms that are mild, transient or chronic to compel healthcare-seeking [14, 16]. It has been proposed that symptoms which impede one’s ability to perform activities of daily living may also compel healthcare-seeking [14]. Taken together, Leventhal’s model suggests that for patients with newly emerging PPS (focus of our study), co-occurrence of increased physical symptom burden with reduced health function may be an underappreciated driver of health care utilization. A more thorough understanding of the intersecting factors which lead patients with newly emerging PPS to initiate care, would allow providers to tailor care so as to better address patients’ concerns, while reducing unnecessary appointments focused on determining symptom causation.

Considering the vicious cycle of symptom occurrence, inactivity, deconditioning, and subsequent worsening of symptoms and disability [17], a critical time for effective health care is at inception of PPS. Despite this, no study has followed patients at the development of PPS to understand when health care utilization increases and what types of health care are received at onset. Understanding the factors that drive health care utilization at onset of PPS is important as health care utilization tends to be elevated at onset of PPS [7] and once health care utilization patterns are established, they can become entrenched [18]. Furthermore, receipt of inappropriate care at the onset of PPS may contribute to PPS becoming chronic [19].

The aim of the current study was to examine interrelationships between physical symptom burden, physical and mental health function (the ability to conduct normal daily activities and fulfill usual roles), and medical and mental health care utilization. Since combat deployment is a known risk factor for developing PPS [20, 21], our sample of Operation Enduring/Iraqi Freedom (OEF/OIF) U.S. military personnel assessed before and after combat deployment provided a unique opportunity to test our hypothesis among individuals with newly emerging persistent physical symptoms. We hypothesized that among individuals with newly emerging persistent physical symptoms, co-occurring increased physical symptom burden and lower health function would be a significant predictor of increased health care utilization.

## Methods

### Protocol

The HEROES study is a prospective longitudinal observational cohort study of U.S. soldiers who were preparing for deployment to Iraq or Afghanistan at Fort Dix, NJ or Camp Shelby, MS. The HEROES project collected self-reported data from 4 time points: pre-deployment (Phase 1, 2005–2008), immediate post-deployment (Phase 2, 2007–2009), 3 months after return from deployment (Phase 3, 2007–2010), and 1 year after return (Phase 4, 2008–2011). The HEROES study initially recruited 790 Army National Guard and Army Reserve Enlisted Soldiers (ages 18–60 years) at time of their on-base pre-deployment medical processing. Deployments typically lasted 12–13 months. Four hundred twenty-two soldiers (53%) were available for post-deployment assessment and completed Phase 2; 23 declined to continue and 345 could not be located. The protocol was approved by the Veterans Affairs New Jersey Healthcare System Institutional Review Board and study personnel obtained written consent. Additional information on the procedures and methods has been previously published [22].

### Study sample

This current study used self-reported data from two time points: one-year before deployment (pre-deployment) and one-year after deployment (post-deployment). To focus on new symptoms in a relatively healthy cohort, exclusion criteria (pre-deployment) included current depression, taking medications with cardiovascular and/or autonomic effects, history of schizophrenia or bipolar disorder, or current cancer, high blood pressure, or pregnancy.

### Variables

#### Health care utilization

At one-year post-deployment, Veterans were asked about health care utilization including primary care, specialty care, allied health therapy (e.g., physical therapy, occupational therapy) and mental health care. Health care utilization questions were adapted from the National Health Interview Survey (NHIS) [7, 10, 22]. For questions relating to use of specialty care, Veterans were asked how many times they had seen a gastroenterologist, pulmonologist, rheumatologist, neurologist or other specialist; these items were added together for a total specialist score. For each type of utilization, Veterans were asked how many times they had received each type of care in the past year. To address the positive skew of the count items, after data collection these were transformed into an ordinal scale consistent with the National Health Interview Survey (0, 1, 2–3, 4–9, 10 and above visits).

All analyses controlled for pre-deployment utilization, which was assessed with a single item that asked Veterans how many medical appointments he/she had in the past year. This was also transformed into the same ordinal scale.

#### Physical symptoms

Physical symptoms were captured at pre- and post-deployment by the Patient Health Questionnaire-15 (PHQ-15), which asks respondents how burdened they were by physical symptoms over the previous 14 days. Each item was measured on a scale from 0 (not burdened at all) to 2 (burdened a lot) and these were summed for a total burden score (range 0–30). Physical symptom burden was categorized as follows: 15+ (high), 10–14 (medium), 5–9 (low), and 0–4 (none/mild). Consistent with established thresholds [23], medium/high denoted presence of PPS and mild/low/none denoted absence of PPS. Use of the PHQ-15 as a PPS screening instrument has been validated against clinician determination [24–26].

#### Health function

We used the Veteran’s Rand-36 (VR-36) to examine physical as well as mental health function at pre- and post-deployment [27]. The VR-36 provides two composite scores (each ranging from 0 to 100), with higher scores representing better physical or mental health functioning [28]. Composite scores are normed to a mean of 50 and a standard deviation of 10 [29]. Physical health function indicates ability to perform daily physical activities (e.g., walk a mile) as well as limitations in the ability to perform social roles (e.g., work) due to physical health. Mental health function indicates mood, vitality and limitations in the ability to perform social roles due to mental health.

### Statistical analysis

We generated descriptive statistics for all measures, as well as bivariate correlations between the independent and dependent variables. Moderation analyses were conducted using Preacher and Hayes process macro. The process macro, an observed variable ordinary least squares regression path analysis modeling tool, is widely used in health sciences for estimating two and three-way interactions in moderation models along with simple slopes and regions of significance for probing interactions [30]. This macro applies a bootstrapping method where analyses are run by taking multiple samples to estimate the coefficient and 95% confidence interval. Bootstrapping allows for non-normal distribution of the variables and model [30].

In moderation analyses, we ran separate models for each of four dependent variables (primary care, specialty care, allied health therapy, mental health care). Each of the four models controlled for age, gender, baseline frequency of health care utilization, baseline physical symptoms, baseline physical health function and baseline mental health function. Independent variables in all four models included physical symptoms 1 year after deployment, physical health function 1 year after deployment and mental health function 1 year after deployment. Moderation analyses were used to examine the interaction between physical symptoms and physical health function 1 year after deployment; the interaction between physical symptoms and mental health function 1 year after deployment; and the triple interaction between physical symptoms, physical health function, and mental health function 1 year after deployment.

## Results

Veterans were primarily male (89.7%) with an average age of 27.9 years before deployment. Most identified as White (77.2%), with 9% identifying as African American, 6.7% identifying as other race, 2.7% identifying as American Indian and 2.4% identifying as Asian. Most identified as non-Hispanic (87.6%). At baseline, Veterans reported an average of one visit to a doctor’s office in the past 12 months. We report their health care utilization 1 year after deployment in Table 1.

**Table 1:** Number (Percent) Reporting Health Care Utilization One year After deployment

Number of visits	Primary Care	Specialty Care	Allied Health Care	Mental Health Care
0	102 (32.0%)	219 (68.7%)	234 (73.4%)	206 (64.6%)
1	77 (24.1%)	36 (11.3%)	20 (6.30%)	24 (7.50%)
2–3	96 (30.1%)	30 (9.40%)	22 (6.90%)	28 (8.80%)
4–9	32 (10.0%)	23 (7.20%)	14 (4.40%)	30 (9.40%)
10+	12 (3.80%)	11 (3.40%)	28 (8.80%)	31 (9.70%)

As previously reported [31], mean physical component summary score (PCS) and mean mental component summary score (MCS) - reflecting physical and mental health functioning, respectively - decreased from before deployment (PCS = 55.5 [SD: 5.2], MCS = 48.0 [SD: 9.1]) to 1 year after deployment (PCS = 51.5 [SD:8.8], MCS = 44.9 [SD:12.4]). In contrast, physical symptoms increased significantly (*p* < 0.05) from before deployment (5.2 [SD:3.9]) to 1 year after deployment (7.7 [SD:5.4]).

The bivariate relationships between variables are reported in Table 2. As expected, physical symptoms after deployment, and physical health function after deployment, were each related to primary care utilization, specialty care utilization, utilization of allied therapy (e.g., physical therapy) and mental health care utilization. Mental health function after deployment was related to specialty care utilization and mental health care utilization; contrary to expectations, it was not related to primary care utilization or utilization of allied therapy.

**Table 2:** Correlations between health care utilization, physical symptoms and health

	2	3	4	5	6	7	8	9	10	11
1. Primary care after deployment	0.27*	0.33*	0.26*	0.13*	0.08	− 0.10	0.02	0.22*	− 0.27*	− 0.04
2. Specialist after deployment	1.0	0.44*	0.25*	0.09	0.10	−0.15*	0.06	0.31*	−.041*	− 0.11*
3. Therapist after deployment	0.44*	1.00	0.28*	0.09	0.11*	−0.24*	0.07	0.30*	−0.46*	−0.05
4. Mental health after deployment	0.25*	0.28*	1.00	0.05	0.16*	−0.18*	−0.00	0.32*	−0.19*	− 0.30*
5. Health care Utilization before deployment	0.09	0.09	0.05	1.00	0.13*	−0.14*	−0.01	0.07	−0.03	− 0.06
6. Physical symptoms before deployment	0.10	0.11*	0.16*	0.13*	1.00	−0.33*	−0.48*	0.40*	−0.16*	− 0.23*
7. Physical health function before deployment	−0.15*	− 0.24*	−0.18*	− 0.14*	−0.33*	1.00	−0.15*	− 0.17*	0.29*	0.02
8. Mental health function before deployment	0.06	0.07	−0.00	−0.01	− 0.48*	−0.15*	1.00	−0.14*	− 0.06	0.34*
9. Physical symptoms after deployment	0.31*	0.30*	0.32*	0.07	0.40*	−0.17*	−0.14*	1.00	−0.46*	− 0.57*
10. Physical health function after deployment	−0.41*	− 0.46*	−0.19*	− 0.03	−0.16*	0.29*	−0.06	− 0.46*	1.0	− 0.00
11. Mental health function after deployment	−0.11*	− 0.05	−0.30*	− 0.06	−0.23*	0.02	0.34*	−0.57*	−0.00	1.00

*Represents *p* < .05

We ran four models with each type of health care utilization after deployment as the dependent variable (primary care, specialty care, allied therapy and mental health care). The predictor variables were physical symptoms, physical health function and mental health function 1 year after deployment.

**Fig. 1:**
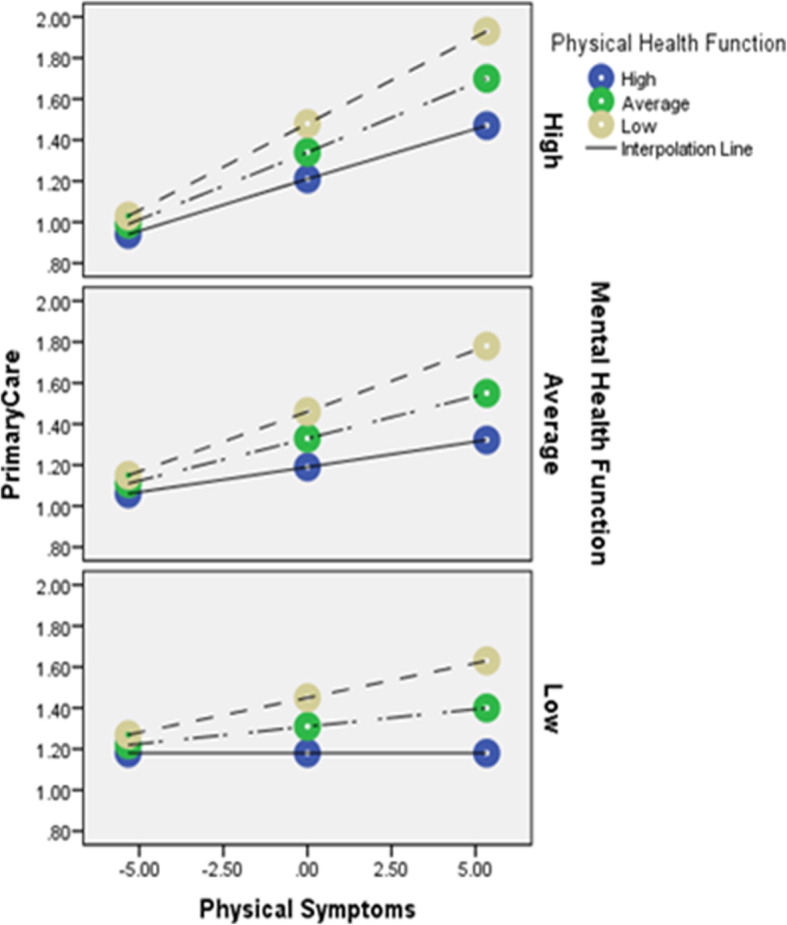
Triple interaction of physical symptoms, physical heath function and mental health function on primary care utilization. Notes x-axis is level of physical symptoms; lines are level of physical health function; panels are levels of mental health function; y-axis is level of primary care utilization

In the first model, 14% of the variability in primary care utilization (*F* = 4.64 [11, 303], *R*^*2*^ = .14, *p* < .001) was explained by the model. There was a trend towards a significant interaction between physical symptoms and physical health function such that physical symptoms best predicted primary care utilization when physical health function was low (*F* = 2.92 [1, 303], *R*^*2*^Δ = .01, *p* = .09) and there was a significant interaction between physical symptoms and mental health function such that physical symptoms best predicted primary care utilization when mental health function was high (*F* = 5.03 [1, 303], *R*^*2*^Δ = .01, *p* = .03). There was also a triple interaction between physical symptoms, physical function and mental health function (*F* = 3.63 [2, 303], *R*^*2*^Δ = .02, *p* = .03), such that physical symptoms best predicted primary care utilization when physical function was low and mental health function was high (Fig. 1).

**Fig. 2:**
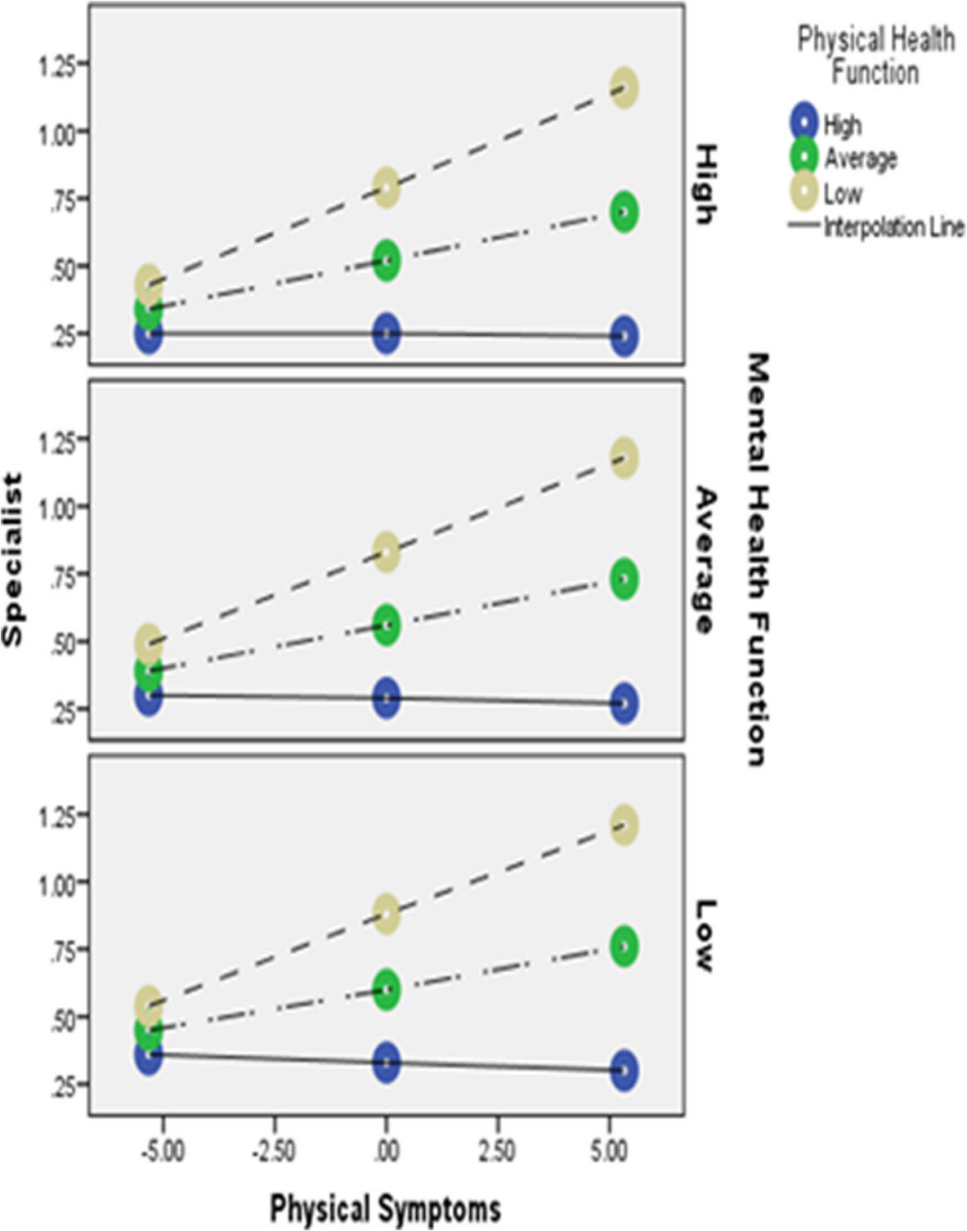
Triple interaction of physical symptoms, physical heath function and mental health function on specialty care utilization. Notes x-axis is level of physical symptoms; lines are level of physical health function; panels are levels of mental health function; y-axis is level of specialty care utilization

In the second model, 24% of the variability in specialty care utilization (*F* = 8.8 [11, 303], *R*^*2*^ = .24, *p* < .001) was explained by the model. Although there was a significant interaction between physical symptoms and physical health function (*F* = 13.61 [1, 303], *R*^*2*^Δ = .03, *p* < .001) (such that physical symptoms best predicted specialty care utilization when physical function was low), we failed to detect a significant interaction between physical symptoms and mental health function (*F* = .06 [1, 303], *R*^*2*^Δ = .00, *p* = .81). Consistent with model 1, there was a significant triple interaction between physical symptoms, physical health function and mental health function (*F* = 6.81 [2, 303], *R*^*2*^Δ = .03, *p* < .001), such that physical symptoms best predicted specialty care utilization when physical health function was low and mental health function was high (Fig. 2).

**Fig. 3:**
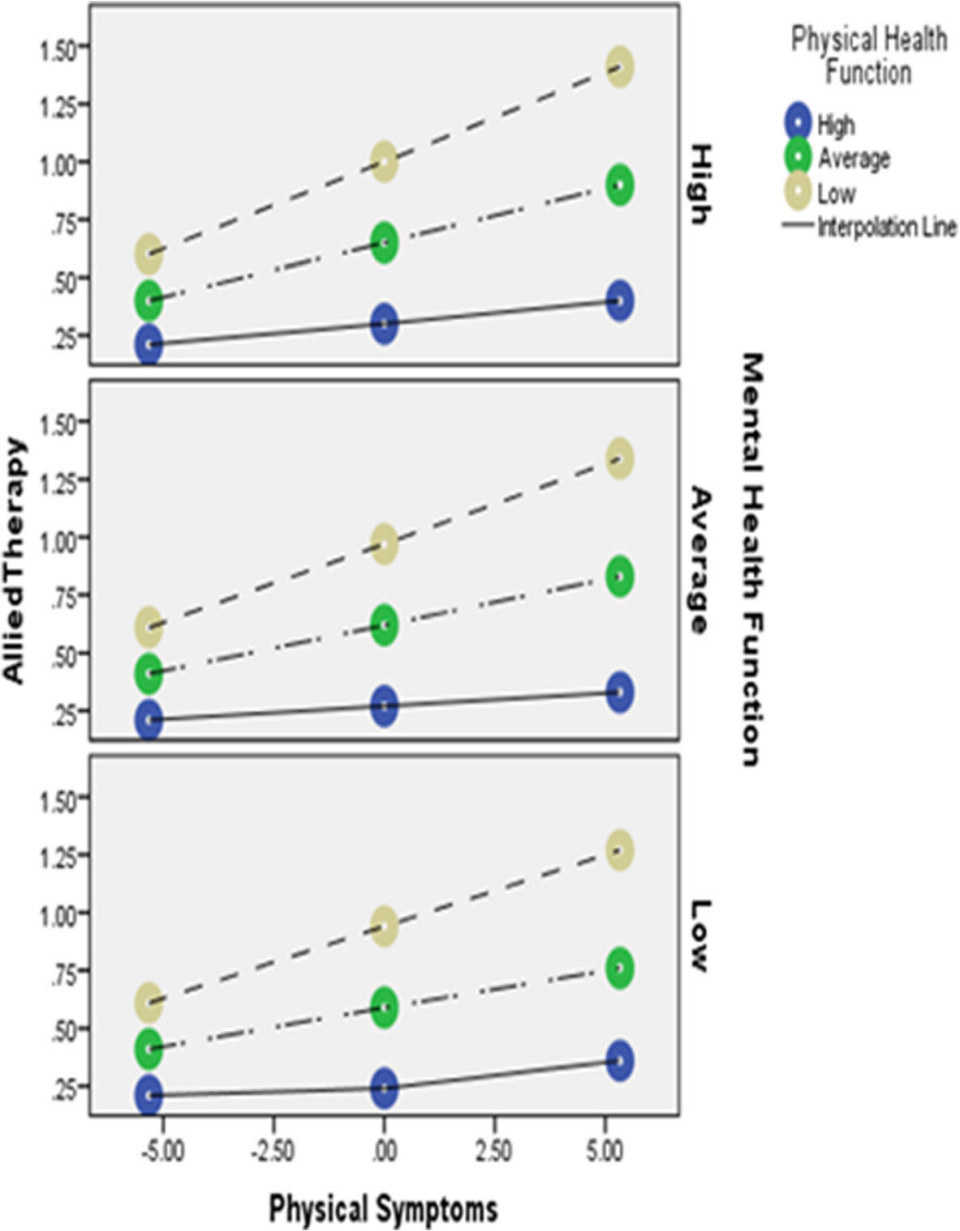
Triple interaction of physical symptoms, physical heath function and mental health function on allied health therapy care utilization. Notes x-axis is level of physical symptoms; lines are level of physical health function; panels are levels of mental health function; y-axis is level of allied health therapy care utilization

In the third model, 27% of the variance in allied therapy utilization (e.g., physical therapy), (*F* = 10.33 [11, 303], *R*^*2*^ = .27, *p* < .001) was predicted by the model. Although there was a significant interaction between physical symptoms and physical health function (*F* = 7.41 [1, 302], *R*^*2*^Δ = .02, *p* = .01) (such that physical symptoms best predicted allied health therapy utilization when physical function was low), we did not detect a significant interaction between physical symptoms and mental health function (*F* = .35 [1, 303], *R*^*2*^Δ = .00, *p* = .55). Consistent with models 1 and 2, there was a significant triple interaction between physical symptoms, physical health function and mental health function (*F* = 3.76 [2, 302], *R*^*2*^Δ = .02, *p* = .02), such that physical symptoms best predicted allied therapy utilization when physical health function was low and mental health function was high (Fig. 3).

In the fourth model (Figure not shown), 18% of the variance in mental health care utilization (*F* = 6.07 [11, 303], *R*^*2*^ = .18, *p* < .001) was predicted by the model, although none of the interaction terms were significant: Physical symptoms and physical health function ((*F* = 2.71 [1, 303], *R*^*2*^Δ = .01, *p* = .10); physical symptoms and mental health function ((*F* = .63 [1, 303], *R*^*2*^Δ = .00, *p* = .43); physical symptoms, physical and mental health function (*F* = 1.82 [1, 303] *R*^*2*^Δ = .01, *p* = .16).

Finally, given fairly high attrition rate, we compared baseline physical symptom severity between those who were or were not lost to follow-up. We found that physical symptom severity at baseline was not significantly (*p* = 0.85) related to the likelihood of being lost to follow-up at one-year post-deployment.

## Discussion

Patients with persistent physical symptoms (PPS) utilize high levels of health care leading to iatrogenic consequences for the patient and significant burden on the health care system. Nevertheless, little is known about what factors trigger patients with PPS to utilize health care, especially at the onset of PPS. We hypothesized that physical symptoms lead to greater health care utilization when co-occurring with poor health function. We tested this hypothesis among U.S. military personnel who were returning from deployment, expecting a sizable portion to develop clinically significant PPS.

As expected, we found that increases in physical symptom burden co-occurring with low physical health function were associated with greater utilization of medical (but not mental health) care services - as compared to greater physical symptom burden co-occurring with average or high physical health functioning. Our findings are consistent with a large literature demonstrating that patients with PPS use disproportionately large amounts of medical health care (but not mental health care) services [3, 4]. Escobar et al., found those with high levels of PPS reported greater use of health services than those with low levels [32]. Barsky et al., found that patients with PPS have approximately twice the outpatient and inpatient utilization and approximately twice the annual health care costs, relative to those without PPS [6]. It was further found that mental health care was the only form of utilization that was not significantly elevated among those with persistent physical symptoms [6]. Our finding that co-occurrence of physical symptoms with functional impairment is a determinant of healthcare-seeking adds to the literature by providing a greater understanding of how symptoms and impairment interact to drive care at the onset of PPS.

Our finding that greater physical symptom burden co-occurring with low physical health function was associated with greater use of medical care services further suggests that Veterans with PPS may be seeking improved health function, rather than merely symptom relief. Historically, medicine has used a symptom-driven approach to care, with patients and providers seeking to understand and treat cause of symptoms rather than impact of those symptoms on health functioning and well-being [33]. However, a symptom-driven approach to medicine can be, as noted by the National Academy of Medicine, inefficient and reactive, particularly for patients with PPS. Our findings suggest that a symptom-driven approach is not only systemically inefficient but also not the main reason patients are seeking care. If health care addresses the factors that lead patients to initiate care, such as low physical health function, as seen in our study, we may better address patients’ concerns, while reducing unnecessary appointments focused on determining cause of symptoms. This shift in focus may also improve the satisfaction of providers, who consider patients presenting with physical symptoms to be among the most challenging to treat [34–36].

Addressing the factors that lead patients to initiate health care is also consistent with medicine’s recent focus on whole health approaches to care delivery. An example of this is the Department of Veterans Affairs (VA), which is undergoing a cultural transformation to focus on “health care that is … driven by what really matters to the person in their life and aligns their health care and goals accordingly” [37]. Improving health function (e.g., reducing limitations in daily activities) and quality of life, and not simply reducing symptoms is a primary goal for many patients seeking health care [10].

In contrast to expectations, our study found that among Veterans with higher physical symptom burden and lower physical health functioning, those with high mental health function used slightly more medical care than those with either average or low mental health functioning. This is an important finding in that it suggests that mental health problems that cause impairments in daily activities and participation (i.e., mental health function) may impede healthcare-seeking, creating barriers to care for this population. We suspect but cannot confirm that patients with poor mental health function may have trouble organizing the many steps it takes to plan, make and attend a health care appointment.

Use of a sample of combat veterans offered strengths and limitations. On the one hand, since combat deployment is associated with PPS, observing service members pre- and post-deployment provided a unique opportunity to observe subjects as they developed significant physical symptom burden. On the other hand, service-members often move after deployment which contributed to the high attrition rate. Nevertheless, concern about high attrition was mitigated by the fact that physical symptom severity at baseline was not significantly related to the likelihood of being lost to follow-up at one-year post-deployment. Additionally, while combat veterans comprise an important population at-risk of PPS, we do not know the generalizability of our results to the civilian population.

Another consideration is that our sample of participants with new PPS consisted mostly of men. Consequently, the extent to which our findings - co-occurring high physical symptom burden with low physical health function was associated with greater medical care use - apply to females cannot be resolved by this analysis. For one thing, persistent physical symptoms are more prevalent among women than men in the general population [38]. For another thing, healthcare-seeking behaviors differ between men and women: for instance, while males seek health care largely in response to physical health concerns, females by contrast often seek care in response to both physical and mental health concerns [39]. Given these and other gender-based differences, follow-up studies of females with new PPS would make a valuable contribution to emerging research.

As additional limitations: (1) we did not have physician diagnosis of PPS. While the PHQ-15 is a common screening measure of PPS, our study would be strengthened by physician corroboration of the unexplained nature of the symptoms; (2) this study also did not capture quality of care. It is therefore unknown if soldiers were receiving appropriate treatments (e.g., cognitive-behavioral therapy) or less appropriate treatments (e.g., opioids) or whether they perceived their care as patient-centered; (3) health care utilization was self-reported raising the possibility of inaccurate or incomplete recollection. The questions used were derived from other well-established and validated surveys, however; (4) while social determinants of health are associated with persistent physical symptoms, we were unable to examine them in this study – subsequent research into the role of social determinants in newly-developing persistent physical symptoms would make a valuable contribution to the research literature.

## Conclusions

The current study found that among Veterans with newly emerging PPS, physical symptom burden is associated with greater medical (but not mental) health care utilization when it co-occurs with poor physical health function. In contrast, poor mental health function was associated with lower health care utilization, suggesting a barrier to access. Taken together, these findings support whole health initiatives that focus on addressing the decrements in health function and well-being experienced by the patient instead of discrete symptoms. Helping patients meet their goals for improving health function, may ultimately improve the efficiency of health care.

## Data Availability

The data that support the findings of this study are available from Veterans Affairs, but restrictions apply to the availability of these data, and so are not publicly available. Data are however available from the authors upon reasonable request and with permission of the Veterans Affairs.

## Abbreviations

PPS: Persistent physical symptoms
OEF: Operation enduring freedom
OI: Operation Iraqi freedom
NHIS: National health interview study
PHQ15: Patient health questionnaire 15
VR36: Veterans rand 36
MCS: Mental component summary score
PCS: Physical component summary score
SD: Standard deviation
VA: Department of veterans affairs
VHA: Veterans health administration
MUS: Medically unexplained symptoms
